# USP5 Promotes Head and Neck Squamous Cell Carcinoma Progression via mTOR Signaling Pathway

**DOI:** 10.1002/cam4.70752

**Published:** 2025-03-11

**Authors:** Ni Xiong, Yue Wang, Junhong Jiang

**Affiliations:** ^1^ Hospital of Stomatology, Guangdong Provincial Key Laboratory of Stomatology Guanghua School of Stomatology, Sun Yat‐Sen University Guangzhou China; ^2^ Department of Stomatology Jiangxi Provincial People's Hospital, The First Affiliated Hospital of Nanchang Medical College Nanchang China

**Keywords:** HNSCC, mTORC1 pathway, prognostic biomarker, USP5

## Abstract

**Background:**

Head and neck squamous cell carcinoma (HNSCC) is a highly aggressive malignancy characterized by limited prognostic markers and treatment options, contributing to high mortality rates. While Ubiquitin‐specific peptidase 5 (USP5) has been implicated in various cancers, its role in HNSCC remains poorly understood.

**Aims:**

This study aims to investigate the role of USP5 in the progression of HNSCC and explore its potential as both a prognostic biomarker and a therapeutic target.

**Materials & Methods:**

This work utilized single‐cell transcriptomic analysis with the Scissor algorithm to identify distinct epithelial subpopulations, particularly focusing on the Stress subpopulation that exhibited significant upregulation of USP5. Validation was conducted using tissue microarray (TMA) analysis and immunohistochemistry (IHC) to compare USP5 expression levels in HNSCC tissues versus adjacent normal tissues. Furthermore, RNA interference (RNAi) experiments were performed to knock down USP5 expression, assessing its effects on tumor cell behavior, including proliferation, migration, and invasion, as well as the regulation of mTORC1 and NF‐κB signaling pathways.

**Results:**

This study revealed that the Stress subpopulation, characterized by USP5 upregulation, was associated with enhanced tumor cell proliferation, migration, and invasion. TMA and IHC analyses confirmed that USP5 expression was significantly higher in HNSCC tissues compared to normal tissues, correlating with poor patient prognosis. Additionally, RNAi‐mediated knockdown of USP5 led to reduced tumor cell activities and downregulation of the mTORC1 and NF‐κB signaling pathways.

**Discussion:**

The findings suggest that USP5 plays a critical role in driving HNSCC progression. Its overexpression in aggressive tumor subpopulations and association with poor clinical outcomes highlight its potential utility as both a prognostic biomarker and a therapeutic target. The observed effects on cell behavior and oncogenic signaling pathways provide mechanistic insights into how USP5 for HNSCC therapy.

**Conclusions:**

This study establishes USP5 as a key driver of HNSCC progression, underscoring its potential role in prognosis and therapy. Targeting USP5 may offer novel treatment strategies for HNSCC, addressing the urgent need for effective therapeutic interventions in this aggressive malignancy.

**Abbreviations**
AKT: protein kinase B
ATP: adenosine triphosphate
EMT: epithelial‐mesenchymal transition
GEO: Gene Expression Omnibus
HNSCC: head and neck squamous cell carcinoma
IHC: immunohistochemistry
KEGG: Kyoto Encyclopedia of Genes and Genomes
mTORC1: mechanistic target of rapamycin complex 1
NF‐κB: nuclear factor kappa‐light‐chain‐enhancer of activated B cells
PI3K: phosphoinositide 3‐kinase
PTEN: phosphatase and tensin homolog
RNAi: RNA interference
ROS: reactive oxygen species
scRNA‐seq: single‐cell RNA sequencing
siRNA: small interfering RNA
TCGA: The Cancer Genome Atlas
TGF‐β: transforming growth factor beta
TMA: tissue microarray
TP53: tumor protein p53
UMAP: uniform manifold approximation and projection
USP5: ubiquitin‐specific peptidase 5

## 1 Introduction

Head and neck squamous cell carcinoma (HNSCC) is one of the most prevalent malignancies within the head and neck region, predominantly affecting the oral cavity, pharynx, and larynx. Globally, HNSCC accounts for over 900,000 new cases annually, with an estimated mortality of approximately 450,000 deaths per year [1, 2]. The clinical management of HNSCC is particularly challenging due to its aggressive nature, characterized by a high propensity for early regional and distant metastasis. This biological behavior is a major contributor to the persistently poor survival rates observed in affected patients. Despite advancements in surgical techniques, radiation therapy, and chemotherapeutic regimens, the overall 5‐year survival rate for HNSCC has seen only marginal improvements over the past few decades [3, 4, 5, 6]. Therefore, identifying novel and effective molecular biomarkers is essential for improving the early diagnosis, prognosis, and treatment of HNSCC.

The epidemiology of HNSCC is highly complex and heterogeneous, with gene mutations being the predominant drivers of this malignancy [7, 8, 9, 10]. Notably, mutations or overexpression of genes, such as EGFR, p53, PIK3CA, and Cyclin D1 are frequently observed in HNSCC, closely correlating with tumor aggressiveness and patient prognosis [11, 12, 13, 14]. Amid the search for new molecular markers, ubiquitin‐specific peptidase 5 (USP5) has garnered increasing attention from researchers [15, 16, 17]. USP5, a member of the deubiquitinase family, is critical in regulating the cell cycle, proliferation, and growth [18, 19] It achieves this by removing polyubiquitin chains, thereby modulating protein stability and degradation. Aberrant expression of USP5 has been documented in various cancer types. For example, in breast cancer, USP5 is known to enhance cancer cell growth and migration by stabilizing β‐catenin [20]. In hepatocellular carcinoma, USP5 promotes cell proliferation by deubiquitinating and inhibiting p53‐induced apoptosis [21]. Additionally, USP5 has been shown to facilitate tumor growth in lung cancer through the regulation of the NF‐κB signaling pathway [22]. These findings indicate that USP5 plays a pivotal role in tumorigenesis and cancer progression.

Despite extensive research on USP5 in various cancers, its specific function in HNSCC remains largely unexplored. This study aimed to examine the expression patterns and clinical significance of USP5 in HNSCC patients. Tissue microarrays (TMAs) were constructed from multiple patient samples, and immunohistochemistry (IHC) was employed to evaluate USP5 expression. The correlation between USP5 expression levels and clinical outcomes was then analyzed. Our results indicated that high USP5 expression is linked to poorer prognosis, suggesting a potential role for USP5 in HNSCC progression. To further investigate the functional role of USP5 in HNSCC, a series of in vitro experiments were performed. Using RNA interference (RNAi), we knocked down USP5 in HNSCC cell lines, leading to notable reductions in cell proliferation, migration, and invasion. Western blot analysis further demonstrated that USP5 knockdown significantly inhibited the activity of key signaling pathways, including mTORC1 and NF‐κB.

In summary, this study is the first to systematically examine the expression and function of USP5 in HNSCC. Our results indicate that USP5 may serve as a valuable prognostic marker and therapeutic target in this malignancy. These findings provide a solid foundation for further investigations into the mechanistic role of USP5 in HNSCC and the development of targeted therapies aimed at USP5.

## 2 Results

### 2.1 USP5 Upregulation in Stress Epithelial Subpopulation Is Linked to Malignant Progression in HNSCC

**FIGURE 1:**
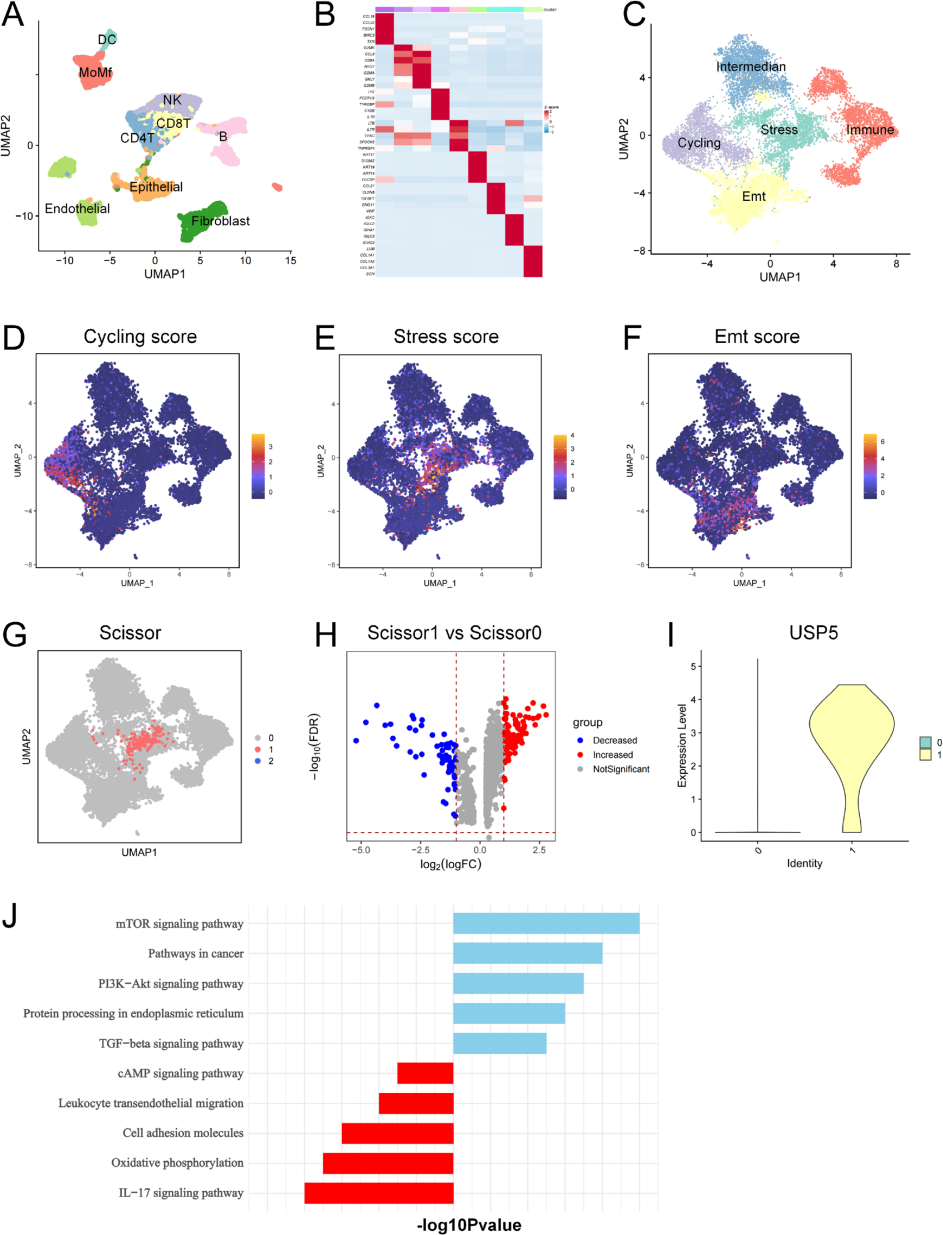
Identification of epithelial subpopulations and their association with malignant progression in HNSCC. (A) UMAP plot showing the clustering of single‐cell RNA sequencing data from HNSCC patients, identifying major cell types, including epithelial, fibroblast, endothelial, and various immune cells. (B) Heatmap displaying the distinct transcriptional profiles of the identified cellular subpopulations. (C) UMAP plot of epithelial cells, further sub‐clustered into five subtypes: Cycling, Stress, EMT, Immune‐associated, and Intermedian epithelial cells. (D–F) Gene set scoring of the Cycling, Stress, and EMT subtypes, respectively, showing the distinct biological features of each subtype. (G) UMAP plot of the Scissor algorithm results, identifying the Stress epithelial subpopulation as being significantly associated with poor survival, using TCGA HNSCC patient data. (H) Volcano plot of differential gene expression analysis between Scissor1 (Stress) and Scissor0 populations, highlighting USP5 as the most significantly upregulated gene in the Stress subpopulation. (I) Violin plot showing the expression levels of USP5 between Scissor1 and Scissor0 populations. (J) KEGG pathway enrichment analysis of differentially expressed genes between Scissor1 and Scissor0 populations, showing pathways enriched in both subpopulations. Notably, pathways associated with tumor progression, such as “mTOR signaling” and “PI3K‐Akt signaling,” were enriched in the Scissor1 population.

Single‐cell RNA sequencing (scRNA‐seq) analysis of HNSCC samples from the GEO database identified distinct cellular subpopulations, including epithelial, fibroblast, endothelial, and various immune cells (Figure 1A). Heatmap clustering of transcriptomic signatures revealed unique gene expression profiles associated with each subpopulation (Figure 1B). Sub‐clustering of epithelial cells further delineated five subtypes: Cycling, Stress, EMT, Immune‐associated, and Intermedian epithelial cells (Figure 1C). Gene set scoring and pathway analysis confirmed that each subtype exhibited unique biological characteristics, with the Stress subpopulation displaying features associated with tumor aggressiveness and poor prognosis (Figure 1D–F). To explore the clinical significance of these epithelial subpopulations, we applied the Scissor algorithm to integrate scRNA‐seq data with patient survival information from TCGA's HNSCC cohort. This analysis identified the Stress epithelial subpopulation as significantly negatively correlated with survival, indicating its potential role in disease progression (Figure 1G). Differential gene expression analysis between the Scissor1 (Stress) and Scissor0 populations revealed that USP5 was the most significantly altered gene, with higher expression in the Scissor1 group (Figure 1H,I). The selection of Scissor1 vs. Scissor0 groups was based on their distinct survival outcomes and contrasting pathway activation patterns, with Scissor1 exhibiting oncogenic signaling enrichment and Scissor0 showing stronger immune response pathways. KEGG pathway enrichment analysis of differentially expressed genes showed that pathways, such as “mTOR signaling,” “PI3K‐Akt signaling,” and “TGF‐beta signaling” were enriched in the Scissor1 population, whereas immune response and cell adhesion pathways were more prominent in the Scissor0 population (Figure 1J). These findings suggest that the Stress subpopulation, characterized by elevated USP5 expression and specific pathway activations, may contribute to the poorer prognosis observed in certain HNSCC cases.

### 2.2 High USP5 Expression is Linked to Poor Overall Survival

**FIGURE 2:**
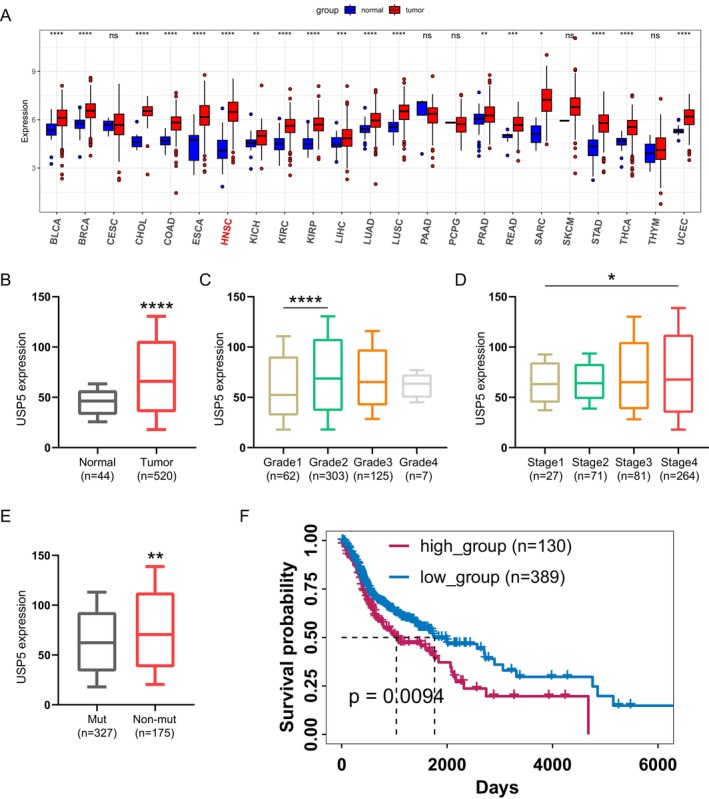
High USP5 expression correlates with poor overall survival in HNSCC. (A) Box plot showing USP5 mRNA expression across 23 different cancer types, comparing tumor (red) and normal (blue) tissues. Statistical significance was assessed using a two‐tailed *t*‐test. ****p < 0.0001, ns, not significant. (B) USP5 mRNA expression in HNSCC tissues compared with normal tissues, analyzed using data from TCGA‐HNSC (****p < 0.0001, two‐tailed *t*‐test). (C) Box plot showing USP5 expression in HNSCC across different tumor grades. Statistical analysis was performed using one‐way ANOVA followed by Tukey's post hoc test (****p < 0.0001). (D) USP5 expression in HNSCC across different cancer stages, with statistical significance determined using one‐way ANOVA followed by Tukey's post hoc test (*p < 0.05). (E) Comparison of USP5 expression between TP53 mutant (Mut) and nonmutant (Non‐mut) HNSCC samples (**p < 0.01, two‐tailed *t*‐test). (F) Kaplan–Meier survival analysis comparing overall survival between patients with high USP5 expression (high_group) and low USP5 expression (low_group) in HNSCC. Statistical significance was determined using the log‐rank (Mantel–Cox) test (*p* = 0.0094).

A thorough pan‐cancer evaluation of USP5 expression across 23 cancer types revealed notable overexpression in 18 of them, including HNSCC (Figure 2A). TCGA‐HNSC data further showed that USP5 mRNA levels are significantly elevated in HNSCC patients compared with normal tissues (Figure 2B). Additionally, USP5 expression was closely associated with more advanced stages, higher tumor grades, and TP53 mutations in HNSCC (Figure 2C–E), underscoring its connection with key pathological factors. Prognostic analysis confirmed that increased USP5 expression correlates with worse clinical outcomes (Figure 2F).

### 2.3 Association of High USP5 Expression With Prognosis in Patient Cohorts

**FIGURE 3:**
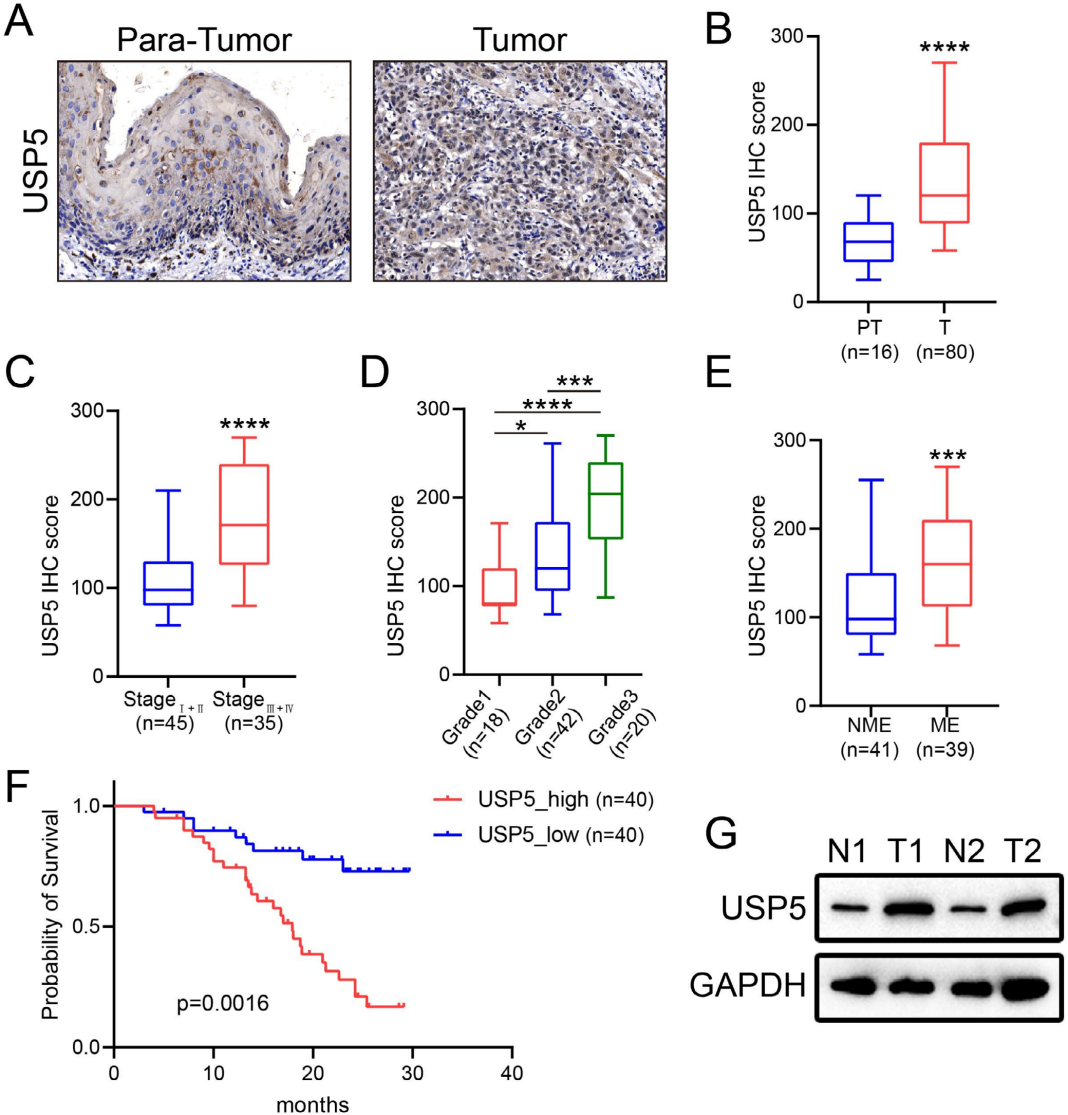
USP5 expression correlates with tumor progression and poor prognosis in patients. (A) Representative images show higher USP5 expression in tumor tissues compared with adjacent non‐tumor tissues. Scale bar, 200 μm. (B–E) Quantitative analysis of USP5 IHC scores across patient subgroups: (B) tumor vs. para‐tumor tissues, (C) disease stages, (D) histological grades, (E) lymph node metastasis status. Statistical analysis was performed using Student's *t*‐test for two‐group comparisons (B) and one‐way ANOVA for multiple group comparisons (C–E). ****p < 0.0001, ***p < 0.001, *p < 0.05. (F) Kaplan–Meier analysis comparing overall survival between patients with high (USP5_high, *n* = 40) and low (USP5_low, *n* = 40) USP5 expression. Statistical significance was determined using the log‐rank (Mantel–Cox) test. (G) Western blot analysis of USP5 protein levels in paired normal (N) and tumor (T) gastric cancer tissue samples. GAPDH was used as a loading control.

In our cohort, USP5 expression was assessed through immunohistochemistry (IHC) (Figure 3A), revealing significant differences in intensity across various groups (Figure 3B). Elevated USP5 expression was particularly noted in patients with more advanced stages and higher histological grades (Figure 3C,D). Additionally, high USP5 levels were strongly linked to lymph node metastasis (Figure 3E), suggesting a more aggressive disease phenotype. Patients with increased USP5 expression had significantly poorer overall survival compared to those with lower expression levels (Figure 3F). Furthermore, we evaluated USP5 expression in two paired gastric cancer tissue samples using western blotting, which confirmed that USP5 protein levels were markedly higher in tumor tissues than in normal ones (Figure 3G). These results emphasize USP5's potential as a prognostic marker, especially for identifying patients at elevated risk of disease progression and metastasis.

### 2.4 USP5 Expression and Functional Role in HNSCC Cell Lines

**FIGURE 4:**
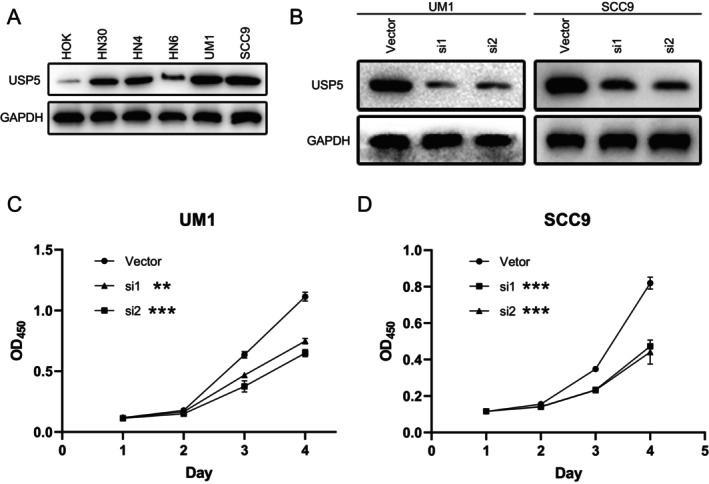
USP5 promotes the proliferation of tumor cells in HNSCC. (A) Western blot analysis of USP5 protein levels across various HNSCC cell lines, including the normal oral epithelial cell line HOK. (B) USP5 protein levels in UM1 and SCC9 cell lines following transfection with USP5‐specific siRNAs (si1 and si2) or control vector. (D) Cell proliferation assays of UM1 (C) and SCC9 (D) cell lines following USP5 knockdown with siRNAs (si1, si2) compared with control vector. *p* values were presented by one‐way ANOVA, Dunnett's test. **p < 0.01, ***p < 0.001.

**FIGURE 5:**
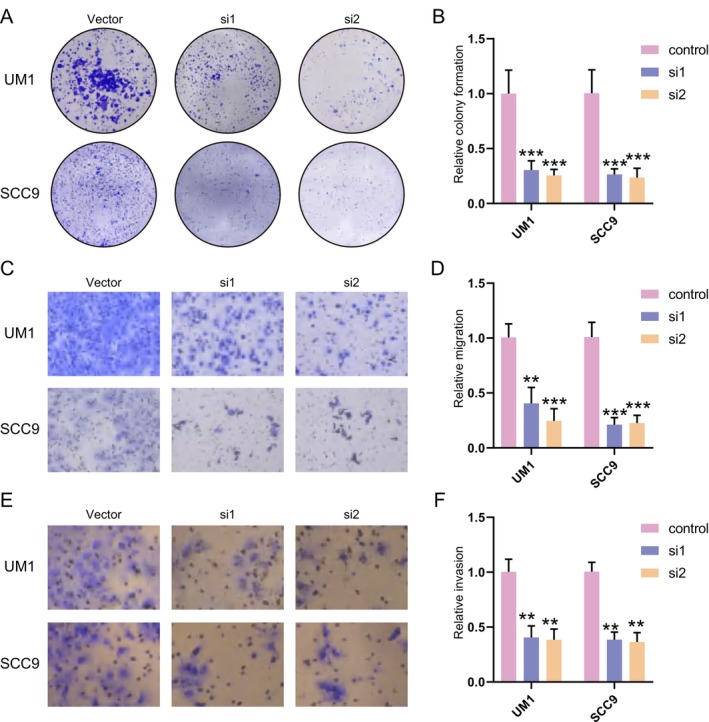
USP5 knockdown impacts colony formation, migration, and invasion in HNSCC cell lines. (A and B) Colony formation assays in UM1 and SCC9 cells following USP5 knockdown with siRNAs (si1, si2) compared with the control vector. Quantification of colony formation is shown in (B) (***p < 0.001). *p* values were presented by one‐way ANOVA with Tukey's multiple comparison test. (C and D) Cell migration assays in UM1 and SCC9 cells 48 h post‐transfection with USP5‐specific siRNAs (si1, si2) or control vector. Quantification of migration is shown in (D) (**p < 0.01, ***p < 0.001). *p* values were presented by one‐way ANOVA with Tukey's multiple comparison test. (E and F) Cell invasion assays in UM1 and SCC9 cells 48 h post‐transfection with USP5‐specific siRNAs (si1, si2) or the control vector. Quantification of invasion is shown in (F) (**p < 0.01). *p* values were presented by one‐way ANOVA with Tukey's multiple comparison test.

We evaluated USP5 protein levels in several HNSCC cell lines, along with the normal oral epithelial cell line HOK. Western blot analysis showed high USP5 expression in UM1 and SCC9, moderate levels in HN30 and HN4, and minimal expression in HOK and HN6 (Figure 4A). We subsequently focused on UM1 and SCC9 for further experiments. To investigate the functional consequences of USP5 depletion, we performed siRNA‐mediated knockdown in UM1 and SCC9 cells. Western blot analysis confirmed efficient USP5 silencing (Figure 4B). Cell proliferation assays demonstrated that USP5 silencing inhibited cell growth (Figure 4C,D). Colony formation assays further revealed a significant reduction in colony formation upon USP5 knockdown in UM1 and SCC9, compared with the control (Figure 5A,B), highlighting USP5's crucial role in promoting HNSCC cell proliferation. Additionally, USP5 knockdown markedly suppressed cell migration and invasion in UM1 and SCC9 48 h after transfection (Figure 5C–F), emphasizing USP5's role in enhancing the migratory and invasive behavior of HNSCC cells.

### 2.5 Differential Gene Expression and Signatures Associated With USP5 Overexpression

**FIGURE 6:**
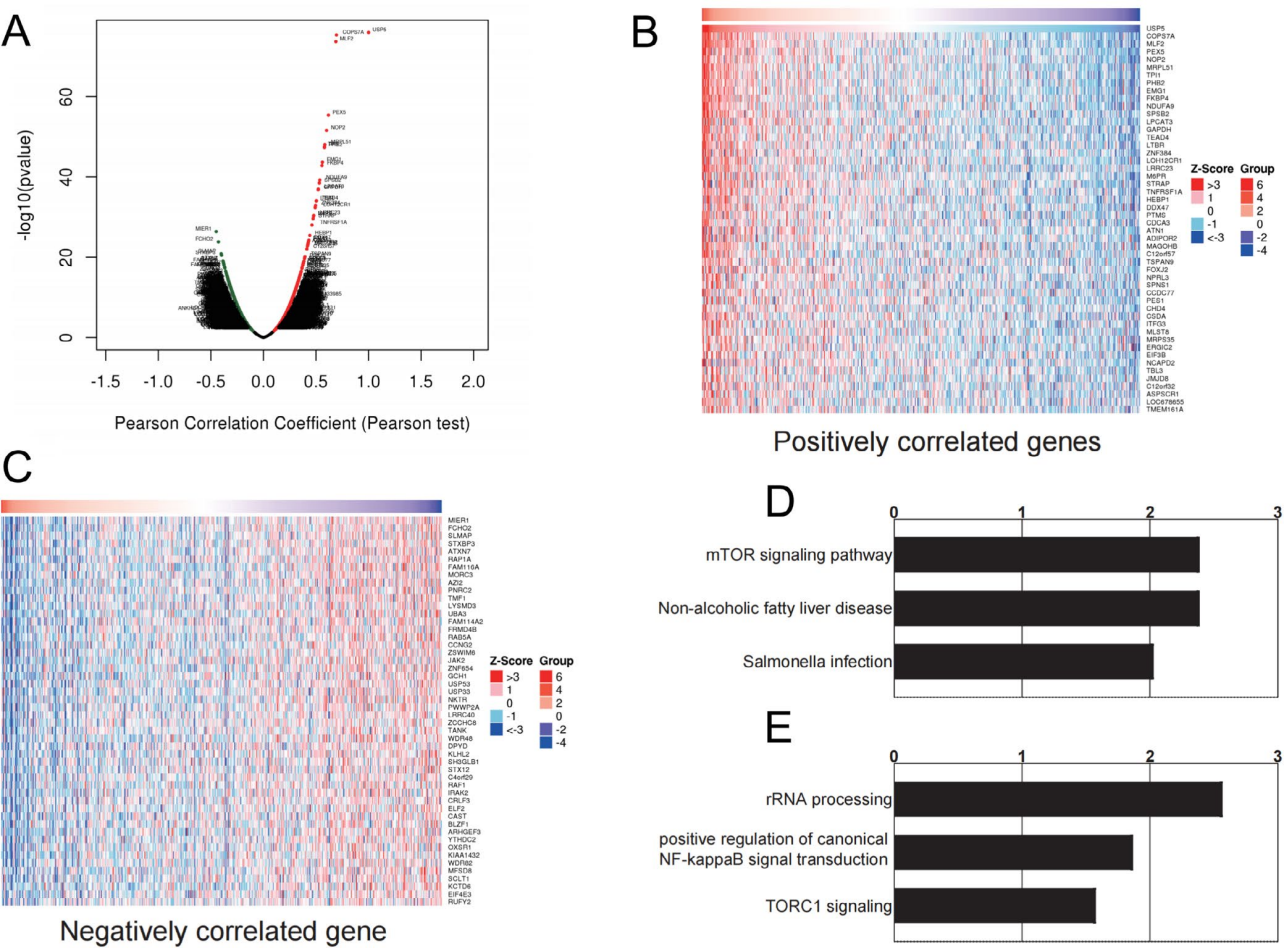
Correlation between USP5 expression and gene expression profiles in HNSCC. (A) Volcano plot illustrating differentially expressed genes associated with increased USP5 expression in the TCGA‐HNSC dataset, identified through Pearson correlation analysis. (B and C) Heatmaps showing the top 50 genes positively (B) and negatively (C) correlated with USP5 expression. (D and E) KEGG pathway enrichment analysis of genes positively (D) and negatively (E) correlated with USP5 overexpression.

Differentially expressed genes linked to elevated USP5 expression were identified from the TCGA‐HNSC database and visualized through a volcano plot (Figure 6A). A heatmap of the top 50 genes positively and negatively correlated with USP5 expression was generated, offering a detailed view of the gene expression profile (Figure 6B,C). Enrichment analysis of overexpressed genes associated with USP5 revealed significant activation of tumorigenic processes, indicating the involvement of multiple oncogenic pathways. Notably, USP5 expression was strongly correlated with the mTOR signaling pathway, nonalcoholic fatty liver disease, and salmonella infection (Figure 6D). In line with these findings, KEGG pathway analysis confirmed a connection between USP5 overexpression and both NF‐κB signal transduction and TORC1 signaling pathways (Figure 6E). These associations suggest that USP5 plays a key role in promoting tumor growth and survival while simultaneously inhibiting tumor‐suppressive mechanisms.

### 2.6 USP5 Knockdown Reduces Activity in Tumorigenic Pathways

**FIGURE 7:**
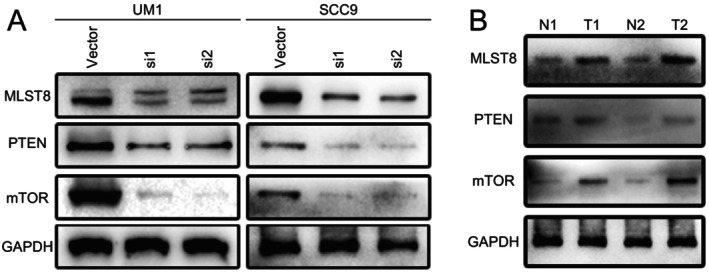
USP5 knockdown suppresses the mTOR signaling pathway. (A) Western blot analysis of MLST8, PTEN, and mTOR protein levels in UM1 and SCC9 cells following USP5 knockdown with siRNAs (si1 and si2) compared with the control vector. (B)Western blot analysis of MLST8, PTEN, and mTOR protein levels in paired normal (N) and tumor (T) tissues from HNSCC patients.

We explored the molecular mechanisms underlying USP5 knockdown and its impact on tumorigenic pathways. Notably, silencing USP5 in UM1 and SCC9 cell lines led to a significant reduction in key proteins involved in the mTOR signaling pathway (Figure 7A). Western blot analysis of patient samples also reflected these findings, showing higher levels of mTOR pathway proteins in tumors compared with normal tissues (Figure 7B). These results further reinforce the critical role of USP5 in regulating mTOR signaling and its potential influence on the aggressiveness of HNSCC.

### 2.7 Single‐Cell RNA Sequencing Data Reveals USP5's Role in HNSCC

**FIGURE 8:**
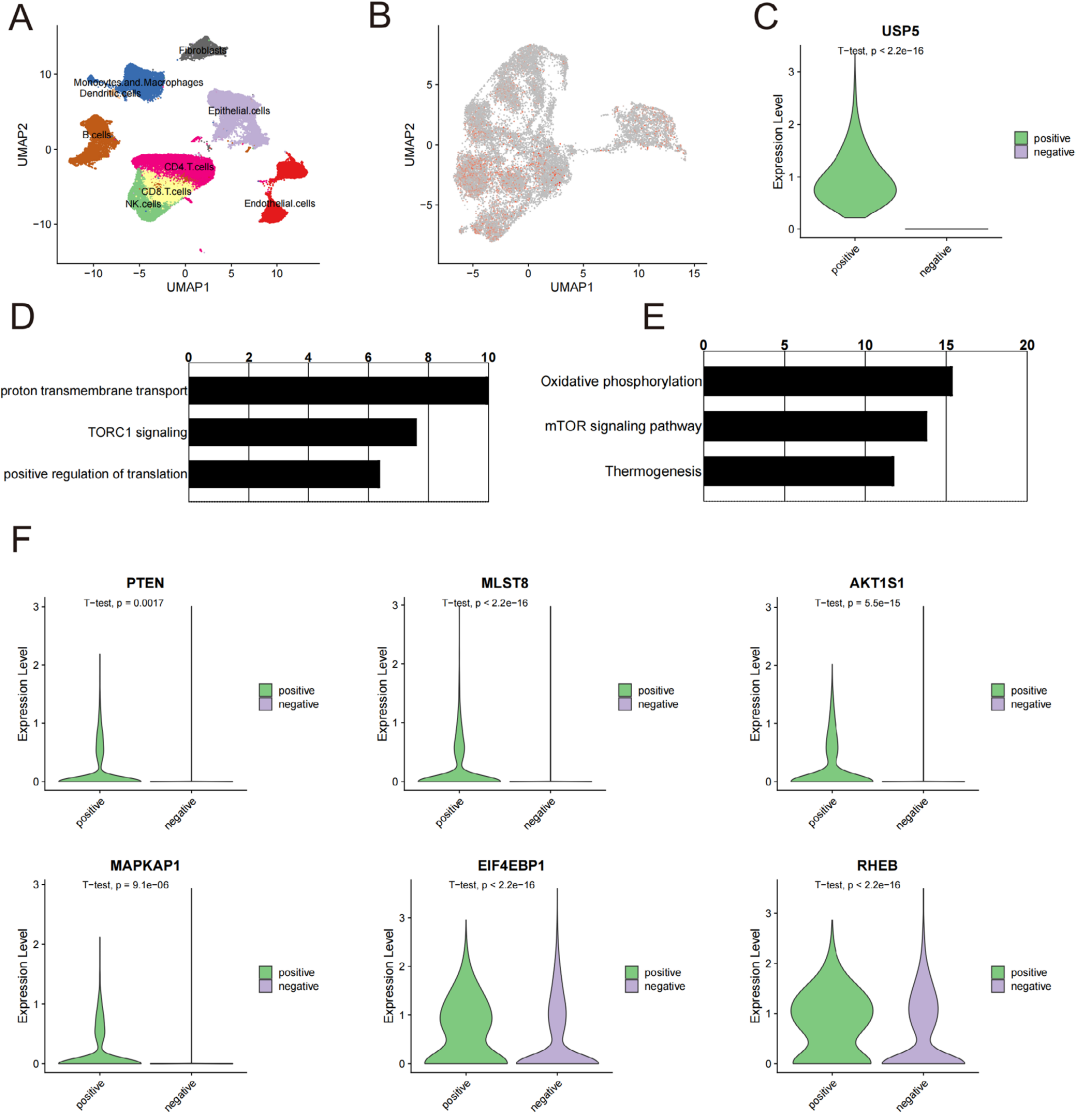
Single‐cell RNA sequencing analysis of USP5 expression in HNSCC. (A) UMAP plot showing the distribution of various cell subpopulations, including tumor epithelial cells, immune cells, fibroblasts, and endothelial cells, in the single‐cell RNA‐seq dataset (GSE164690). (B) UMAP plot highlighting USP5 expression across the cell subpopulations, with predominant expression in tumor epithelial cells. (C) Violin plot comparing USP5 expression levels between USP5‐positive and USP5‐negative epithelial cells. (D and E) Pathway enrichment analysis of differentially expressed genes in USP5‐positive epithelial cells, showing significant involvement in the TORC1 signaling pathway and other related pathways. (F) Violin plots displaying the expression levels of key mTOR pathway genes (PTEN, MLST8, AKT1S1, MAPKAP1, EIF4EBP1, and RHEB) in USP5‐positive versus USP5‐negative epithelial cells.

Finally, we validated USP5 expression at the single‐cell level using a GEO single‐cell dataset (GSE164690). The dataset included several cell subpopulations, including tumor epithelial cells, immune cells, fibroblasts, and endothelial cells (Figure 8A). Notably, USP5 expression was predominantly found in tumor epithelial cells, indicating its significant role in this particular cell type (Figure 8B). We further divided the epithelial cells into USP5‐positive and USP5‐negative groups (Figure 8C) and conducted differential gene expression and enrichment analyses. Enrichment analysis of USP5‐associated genes further supports these findings, indicating that changes in USP5 expression levels significantly impact the mTOR signaling pathway (Figure 8D,E).

Comparative analysis of key mTOR pathway genes between USP5‐positive and USP5‐negative epithelial cells revealed that USP5‐positive cells had higher expression levels of these critical genes (Figure 8F). These alterations suggest that USP5 plays a multifaceted role in promoting tumor progression and metastasis. These insights provide a robust basis for further investigation into the mechanistic pathways involving USP5 and the development of targeted therapies aimed at modulating its expression and activity.

## 3 Discussion

In this study, we applied the Scissor algorithm to identify clinically relevant epithelial subpopulations in HNSCC. Among them, the Stress subpopulation emerged as a distinct subset characterized by significant USP5 upregulation, which was strongly associated with tumor progression and poor prognosis. This subpopulation exhibited enrichment in oncogenic pathways, such as mTOR and PI3K‐Akt signaling, both of which are known to drive tumor growth, survival, and resistance to therapy [6]. The biological relevance of stress‐related epithelial subpopulations has been reported in other malignancies, where cells exhibiting stress‐adaptive phenotypes contribute to increased plasticity, metastasis, and therapy resistance [23, 24]. Our findings suggest that a similar mechanism may be at play in HNSCC, where USP5‐driven signaling enhances the oncogenic potential of the Stress subpopulation. Given the strong association between USP5 expression, oncogenic signaling activation, and poor clinical outcomes, our results suggest that targeting USP5 and its downstream pathways could be a promising therapeutic strategy to mitigate the malignant potential of this subpopulation in HNSCC. Further analysis using the pan‐cancer database revealed USP5 overexpression in 18 out of 23 cancer types, except for acute myeloid leukemia (AML) and skin cancer, when compared with normal tissues. Recent evidence suggests that USP5 supports mTOR signaling integrity and facilitates tumor progression, consistent with our findings that suggest USP5 functions as an oncogene, enhancing tumor cell proliferation and metastasis. Additionally, enrichment analysis of the TCGA‐HNSC database revealed a strong association between USP5 overexpression and increased tumorigenic activity in HNSCC, marked by a positive correlation with proliferation marker genes and a negative correlation with apoptosis‐related genes.

We further assessed the clinical relevance of USP5 protein expression in a cohort of HNSCC patients. Our analysis showed that USP5 levels were significantly elevated in HNSCC tissues compared with adjacent normal tissues. USP5 is a component of the regulatory complex associated with the mTORC1 signaling pathway, which is known to be frequently dysregulated in various cancers, including HNSCC. mTORC1 activity influences the growth, proliferation, and metabolism of HNSCC cells [25, 26]. Our findings indicate that USP5 plays a significant role in cancer progression and is associated with clinical outcomes in patients. HNSCC patients with elevated USP5 expression exhibited markedly lower overall survival compared to those with low expression levels. Notably, our study underscores the prognostic importance of USP5 when combined with common genomic alterations, such as PTEN loss and ERG gain. After adjusting for Gleason grade groups, the results support the potential use of combined biomarkers to classify HNSCC patients into distinct risk categories for predicting lethal disease and overall survival. The additional prognostic impact of PTEN loss and high USP5 expression likely corresponds to increased activity of the PI3K/AKT/mTOR pathway in HNSCC. Furthermore, ERG alterations may influence mTOR signaling and contribute to the development of castration‐resistant disease in this context. Accumulating evidence suggests that USP5 is a critical protein in the mTOR and possibly MAPK pathways, although further research is needed [27]. The mTOR pathway plays an important role in cellular homeostasis and significantly controls cell survival [23]. This could be due to its function as a scaffold protein and its involvement in various cell proliferation pathways. These findings indicate that USP5 is likely involved in tumorigenesis.

To explore the function of USP5 in HNSCC in vitro, we screened various HNSCC cell lines for USP5 protein expression to identify suitable models. The results revealed moderate‐to‐high USP5 expression in most HNSCC cell lines, including HN30, HN4, UM1, and SCC9, whereas the normal oral epithelial cell line HOK exhibited low expression levels. Knockdown of USP5 in UM1 and SCC9 cells significantly decreased cell proliferation, as indicated by reduced colony formation compared with controls. Additionally, we assessed the impact of USP5 on metastasis using migration and invasion assays. Knockdown of USP5 in DU145 cells significantly impaired both cell migration and invasion. These results suggest that USP5 contributes to the metastatic potential of HNSCC cell lines.

In HNSCC, USP5 knockdown led to downregulation of β‐catenin, a key protein in cell adhesion and gene transcription, and upregulation of phosphorylated PTEN, a tumor suppressor. The reduction in β‐catenin levels could impair the cell proliferation and survival pathways commonly activated in cancer cells [28, 29]. Concurrently, the increase in phosphorylated PTEN suggests enhanced PTEN activity, which negatively regulates the PI3K/Akt/mTOR pathway, thereby inhibiting mTOR signaling and potentially reducing tumor growth and progression [30]. This dual effect indicates that USP5 knockdown exerts anti‐tumor effects by modulating these critical molecular pathways.

We further validated USP5 expression at the single‐cell level in HNSCC. Single‐cell analysis revealed that USP5 expression across different cell populations was linked to various key signaling pathways. Notably, cells with elevated USP5 expression showed a strong association with the mTORC1 signaling pathway. These single‐cell findings underscore the critical role of USP5 in HNSCC, supporting its potential as both a prognostic marker and therapeutic target. Altered USP5 expression levels not only influenced tumor cell proliferation and migration but also had significant effects on cell cycle regulation and epithelial–mesenchymal transition (EMT). These results lay a foundation for deeper investigation into the mechanisms by which USP5 drives HNSCC progression and the development of targeted therapies against USP5.

Although our study identifies USP5 as an oncogenic driver in HNSCC, several limitations should be acknowledged. First, the clinical relevance of USP5 overexpression in current HNSCC treatment remains unclear. Whether USP5 affects responses to EGFR inhibitors, immunotherapy, or radiotherapy requires further investigation. Additionally, although DUB inhibitors have shown promise in cancer therapy, no specific USP5‐targeting drugs are currently available. Second, other oncogenic pathways, such as PI3K‐AKT, may also contribute to tumor progression, and their interactions with USP5 warrant further study. Although we expanded our patient cohort to improve statistical power, prospective validation is needed to confirm the prognostic and therapeutic potential of USP5 in HNSCC. Future experiments will focus on genetically modified mouse models and pharmacological inhibition of USP5 to validate its oncogenic function and therapeutic potential in HNSCC. Despite these limitations, our findings highlight USP5 as a promising therapeutic target, providing a foundation for future studies.

In sum, our study demonstrates that USP5 is significantly overexpressed in HNSCC, correlating with enhanced tumorigenic processes, including cell proliferation, migration, and invasion. Knockdown of USP5 impairs these processes, highlighting its potential as a prognostic marker and therapeutic target in HNSCC.

## 4 Methods

### 4.1 Study Population and Tissue Microarray Construction

We evaluated USP5 protein expression in 80 HNSCC patients; demographic details are in Table 1. Selection criteria included HNSCC diagnosis from January 2015 to December 2019 at the affiliated Stomatological Hospital of Guanghua School of Stomatology, Sun Yat‐sen University, complete medical records, and consent for research. Exclusion criteria were previous malignancies, concurrent cancers, or neoadjuvant therapy. Specimens were collected under sterile conditions, fixed in 10% neutral‐buffered formalin for 24–48 h, and paraffin‐embedded. A pathologist identified tumor areas for core extraction using a semi‐automatic tissue arrayer. Cores of 1.0 mm diameter were re‐embedded into recipient blocks in a predefined array. Each patient was represented by two tumor cores and adjacent non‐tumor tissue. Sections (4 μm) were prepared for immunohistochemical analysis. Tumor samples were collected with the patients' written informed consent and approved by the Ethics Committee of the affiliated Stomatological Hospital of Guanghua School of Stomatology, Sun Yat‐sen University. Demographic information for all patients is provided in Table 1.

**TABLE 1:** Demographic information for all patients.

No.	Stage	Grade	Status	Months	LN
1	4	3	1	7.933333333	1
2	1	1	0	16.96666667	0
3	1	2	0	25.63333333	0
4	1	2	0	24.53333333	0
5	3	2	1	24.23333333	1
6	4	3	1	7	1
7	1	2	0	19.96666667	0
8	1	2	0	20.06666667	0
9	3	3	0	19.63333333	1
10	1	2	0	18.56666667	1
11	4	3	1	16.76666667	1
12	2	1	0	21.9	0
13	4	2	1	13.8	1
14	4	2	1	13.23333333	1
15	4	3	1	10	1
16	1	2	0	29.66666667	0
17	1	2	0	29.13333333	0
18	1	1	0	28.03333333	0
19	1	2	0	23.73333333	0
20	4	3	1	10	0
21	1	2	1	17.93333333	0
22	1	2	0	17.46666667	1
23	1	2	1	13.23333333	0
24	3	1	0	10	0
25	1	2	0	25.46666667	1
26	2	2	0	25.6	1
27	1	2	0	16.3	1
28	1	3	0	26.76666667	1
29	1	2	0	22.9	1
30	1	3	1	20.96666667	1
31	2	1	0	24.76666667	0
32	2	2	0	16.26666667	1
33	4	3	0	24.53333333	1
34	2	1	0	23.53333333	0
35	2	2	0	23.3	0
36	3	2	1	21.26666667	0
37	2	2	0	13.73333333	0
38	1	1	0	13.33333333	0
39	1	1	0	11.66666667	0
40	2	2	1	22.6	1
41	2	3	0	29.13333333	1
42	4	3	1	13.5	1
43	2	2	0	26.56666667	0
44	2	2	0	21.86666667	0
45	2	1	1	13.26666667	0
46	3	2	1	25.4	0
47	3	2	1	18.86666667	1
48	3	1	0	28.63333333	1
49	3	1	0	29.36666667	0
50	2	2	0	23	0
51	4	3	1	4.166666667	0
52	2	2	0	26.56666667	1
53	3	2	1	14.4	1
54	2	1	1	12.23333333	0
55	3	2	1	18.73333333	0
56	4	2	0	12.26666667	0
57	3	1	1	18.96666667	1
58	4	2	1	9.566666667	0
59	2	2	0	26.33333333	1
60	2	2	0	19.66666667	1
61	3	2	1	24.23333333	1
62	4	3	1	7	1
63	4	1	0	15.33333333	0
64	3	3	1	17	0
65	2	2	1	11	0
66	3	3	1	4	1
67	1	1	1	23	1
68	2	3	1	8	1
69	4	3	1	3	1
70	2	2	1	7	0
71	4	3	1	16	0
72	2	3	1	18	1
73	4	1	0	13	0
74	3	3	0	13.33333333	1
75	4	2	1	9	1
76	1	1	0	17.66666667	0
77	4	2	0	6.333333333	1
78	4	2	1	8	0
79	1	1	0	5	0
80	2	2	1	14	1

### 4.2 Immunohistochemistry

HNSCC tissues were fixed in neutral‐buffered formalin for 48 h, paraffin‐embedded, and sectioned into 5 mm slices. After dewaxing and hydration, antigen retrieval was performed using Sodium Citrate Antigen Retrieval Solution. Endogenous peroxidase activity was blocked with 3% hydrogen peroxide for 10 min, followed by 30‐min blocking with 5% BSA at 37°C. Sections were incubated overnight at 4°C with USP5 primary antibody (66213‐1‐Ig, Proteintech, 1:200). The next day, a biotinylated secondary antibody was applied at 37°C for 30 min. After PBS washes, a streptavidin‐biotin complex (SABC kit, Boster Biological Technology) was added for 20 min at 37°C. Finally, sections were counterstained with hematoxylin, dehydrated, cleared, and mounted. Digital images were captured using a section scanner after air‐drying overnight.

### 4.3 Pathological Analysis

Staining intensity and the percentage of positively stained cells were evaluated under a light microscope. The H‐score was calculated on a 300‐point scale by multiplying staining intensity (graded 0–3) by the percentage of positive cells. Intensity was scored as 0 (no staining), 1 (weak), 2 (moderate), and 3 (strong). The H‐score formula is: H‐score = (percentage of cells at intensity 1 × 1) + (percentage at intensity 2 × 2) + (percentage at intensity 3 × 3), resulting in a final score from 0 to 300, where higher scores indicate greater protein expression.

### 4.4 Cell Lines and Cell Culture

All cell lines used in this study were purchased from the American Type Culture Collection (ATCC; Manassas, CA, USA) and maintained in our laboratory. HOK cells were cultured in Oral Keratinocyte Medium (OKM, Cat. #2611). HN30, HN4, HN6, and SCC9 cells were cultured in DMEM medium (GIBCO Life Technologies, Grand Island, NY, USA). UM1 cells were cultured in RPMI 1640 medium (GIBCO Life Technologies, Grand Island, NY, USA). All media were supplemented with 10% fetal bovine serum (FBS) (GIBCO Life Technologies, Grand Island, NY, USA) and 1% penicillin–streptomycin (GIBCO Life Technologies, Grand Island, NY, USA). All cells were incubated at 37°C in a 5% CO_2_ environment.

### 4.5 Cell Line Transfection and RNA Interference

USP5 knockdown in HNSCC cell lines was achieved using predesigned USP5 Silencer Select siRNAs (Thermo Fisher), with scramble siRNA as the control. HNSCC cells (SCC9 and UM1) were seeded in 6‐well plates and grown to 70%–90% confluency. Cells were washed with DPBS, and the transfection premix was prepared in Opti‐MEM by combining siRNA and Lipofectamine RNAiMAX (Invitrogen). The siRNA mix was added to each well, and cells were incubated at 37°C with 5% CO_2_. Knockdown efficiency was assessed by western blot at 24‐h intervals up to 96 h.

### 4.6 Western Blot

Proteins from HNSCC cells were lysed in RIPA buffer with protease and phosphatase inhibitors, then centrifuged at 12,000 *g* for 15 min at 4°C. Protein concentration in the supernatant was measured using the Bradford assay. Equal amounts of protein (30 μg per lane) were separated on 10% SDS‐PAGE and transferred to a PVDF membrane at 100 V. The membrane was blocked with 5% nonfat dry milk in TBST for 1 h and incubated overnight at 4°C with primary antibodies specific to USP5 (66213‐1‐Ig, Proteintech, Wuhan, China, diluted 1:1000), mTOR (66888‐1‐Ig, Proteintech, Wuhan, China, diluted 1:1000), PTEN (60300‐1‐Ig, Proteintech, Wuhan, China, diluted 1:1000), MLST8 (A1059, Abconal, guangzhou, China, diluted 1:1000), GAPDH (10494‐1‐AP, Proteintech, Wuhan, China, diluted 1:3000). After washing, it was incubated with HRP‐conjugated secondary antibody (diluted 1:5000) for 1 h at room temperature. Protein bands were detected using ECL and quantified by densitometry, with GAPDH as the loading control. Images were captured and analyzed using an imaging system.

### 4.7 Colony Formation Assay

Briefly, UM1 and SCC9 cells were transfected with either USP5 siRNA or scramble siRNA (control) as described previously. After 24 h, viable cells were stained with trypan blue (Sigma‐Aldrich) and counted using an Olympus automatic cell counter. Around 1000 live cells were plated in 6‐well plates with 1% FBS media and incubated at 37°C with 5% CO_2_ for 10 days. Cells were then fixed, stained with Diff‐Quick, washed with ddH_2_O, and colonies were counted under a stereomicroscope.

### 4.8 Migration and Invasion Assay

UM1 and SCC9 cells were transfected as described earlier and incubated for 24 h at 37°C in a 5% CO_2_ environment. The cells were then trypsinized and counted using an automatic cell counter (Olympus, Breinigsville, PA, USA). Approximately 25,000 cells from each treatment were placed into a 0.8 μm insert Corning BioCoat control inserts (Ref# 354578, Corning, Bedford, MA, USA) for the migration assay, whereas the same number of cells was placed into a Corning Matrigel invasion chamber (Ref# 354480, Corning, Bedford, MA, USA) for the invasion assay. After 24 h, the cells were fixed and stained with Diff‐Quick (Siemens Healthcare Diagnostics, Tarrytown, NY, USA) to prepare the cells for counting. Multiple frames were captured at 10× and 4× magnifications, and the average number of migrated or invaded cells was calculated.

### 4.9 USP5 Expression in the Cancer Genome Atlas TCGA‐HNSC

Initially, USP5 expression across 22 tissue types, including HNSCC, was assessed using pan‐cancer analysis tools and data from TCGA, GTEx, and TARGET databases. Additionally, LinkedOmics (http://www.linkedomics.org, accessed on December 12, 2023) was used in conjunction with the TCGA‐HNSC database to obtain the differentially expressed gene list associated with USP5 overexpression. Finally, we performed an analysis of USP5 gene expression and signature correlations in TCGA‐HNSC. Scatter plots, generated in R, were employed to explore these relationships, with the x‐axis representing log2‐transformed, normalized expression levels (TPM + 1) from RNA‐seq data and the y‐axis displaying various biological pathway signatures and processes of interest.

### 4.10 Single‐Cell Analysis

To further investigate the expression of USP5 at the single‐cell level, we analyzed publicly available HNSCC single‐cell RNA sequencing datasets obtained from the GEO database [GSE164690]. Raw sequencing data were processed using Cell Ranger (10× Genomics) to generate gene expression matrices. Subsequent downstream analyses, including data normalization, principal component analysis (PCA), clustering analysis, and UMAP dimensionality reduction, were performed using the Seurat package (version 3.2.2). Subpopulation analysis of high USP5‐expressing cells was conducted to explore the association of USP5 with specific biological pathways.

### 4.11 Statistical Analysis

Descriptive statistics were reported for the study variables. Means and standard deviations were reported for continuous variables. Frequencies and proportions were reported for categorical variables. Independent t‐tests were used to compare mean values between two groups, and Chi‐square tests were used to compare proportions between categorical variables. Overall survival (OS) and head and neck squamous cell carcinoma‐specific mortality (HNSCC‐SM) were analyzed using Kaplan–Meier estimates. OS was calculated from the date of diagnosis to death due to any cause, with patients alive at the last follow‐up date being censored. HNSCC‐SM was defined as death due to HNSCC, with patients remaining alive or who died due to other reasons being censored. Log‐rank statistics were used to compare the KM curves between groups. Adjusted and unadjusted analyses of OS and HNSCC‐SM were conducted using Cox proportional hazard models. OS and HNSCC‐SM models were adjusted for Gleason score. Hazard ratios (HR) and the corresponding 95% confidence intervals were reported. A *p* value < 0.05 was used for all statistical significance. SPSS version 29 (IBM Corp. (2021). IBM SPSS Statistics for Windows. Armonk, NY, USA: IBM Corp.) was used for all statistical analyses.

## 5 Conclusions

This study identifies USP5 as a critical player in HNSCC progression, with overexpression correlating with poor prognosis and enhanced tumor aggressiveness. USP5 modulates key oncogenic pathways, including mTORC1 and NF‐κB, underscoring its potential as both a prognostic biomarker and a therapeutic target in HNSCC. These findings lay the groundwork for future targeted therapies aimed at USP5 in this malignancy.

## Author Contributions

**Junhong Jiang** and **Ni Xiong:** conceptualization, data analysis and curation. **Yue Wang:** methodology, investigation, validation. **Junhong Jiang:** writing – review and editing, supervision, funding acquisition.

## Ethics Statement

A cohort containing 80 HNSCC specimens was obtained from the affiliated Stomatological Hospital of Guanghua School of Stomatology, Sun Yat‐sen University. The study was conducted in accordance with the Declaration of Helsinki and approved by the Ethics Committee of the affiliated Stomatological Hospital of Guanghua School of Stomatology, Sun Yat‐sen University.

## Consent

Informed consent was obtained from all participants, including consent to publish potentially identifying clinical information. All data in this study were anonymized and processed in compliance with relevant privacy protection regulations.

## Conflicts of Interest

The authors declare no conflicts of interest.

## Data Availability

The single‐cell RNA sequencing datasets analyzed during the current study are available in the GEO repository, https://www.ncbi.nlm.nih.gov/geo/query/acc.cgi?acc=GSE164690. The TCGA datasets analyzed during the current study are publicly available in the TCGA repository, TCGA (https://tcga‐data.nci.nih.gov/tcga/).
